# Metal Imidazole-Modified Covalent Organic Frameworks as Electrocatalysts for Alkaline Oxygen Evolution Reaction

**DOI:** 10.3390/molecules29215076

**Published:** 2024-10-27

**Authors:** Meng Xia, Xinxin Yu, Zhuangzhuang Wu, Yuzhen Zhao, Lijuan Feng, Qi Chen

**Affiliations:** 1 School of Bioengineering, Zhuhai Campus of Zunyi Medical University, Zhuhai 519041, China; xmeng@hainanu.edu.cn; 2 School of Marine Science and Engineering, Hainan University, Haikou 570228, China; xxyu@hainanu.edu.cn (X.Y.); yzzhao@hainanu.edu.cn (Y.Z.); chenqi@hainanu.edu.cn (Q.C.)

**Keywords:** covalent organic frameworks, post-synthetic modification, cobalt coordination, oxygen evolution reaction

## Abstract

Since the product contains no carbon-based substances and can be driven by non-carbon-based electricity, electrocatalytic water splitting is considered to be among the most effective strategies for alleviating the energy crisis and environmental pollution. This process helps lower greenhouse gas emissions while also supporting the shift toward renewable energy sources. The anodic oxygen evolution reaction (OER) involves a more complex multi-electron transfer process, which is the principal limiting factor in overall water splitting. Extensive research has demonstrated that the controlled design of effective electrocatalysts can address this limitation. In this study, a previously unreported covalent organic framework material (COF-IM) was synthesized via a post-synthetic modification strategy. Notably, COF-IM contains imidazole nitrogen metal active sites. Transition metal-coordinated COF-IM@Co can function as a highly effective electrocatalyst, exhibiting a lower overpotential (403.8 mV@10 mA cm^−2^) in alkaline electrolytes, thereby highlighting its potential for practical applications in energy conversion technologies. This study offers new perspectives on the design and synthesis of COFs, while also making substantial contributions to the advancement and application of OER electrocatalysts.

## 1. Introduction

Electrochemical reactions are governed by the disruption and formation of chemical bonds, enabling the conversion of electrical energy into chemical energy and vice versa, allowing the stored chemical energy to be transformed back into electrical energy when needed [1,2]. This bidirectional energy flow is not only pivotal for the efficient operation of energy storage systems but also plays a critical role in stabilizing power grids, especially as we transition to renewable energy sources [3]. These processes are essential for sustainable energy systems, particularly in the context of transitioning to cleaner energy sources, as they offer a means to store excess renewable energy and utilize it during periods of peak demand [4]. One of the most promising methods among these processes is green hydrogen production through electrochemical water oxidation, which stands out as a practical and pollution-free technology that has been aimed at reducing reliance on non-renewable sources since the industrial era [5]. This innovative approach not only facilitates the production of hydrogen as a clean fuel but also integrates seamlessly with renewable energy sources, enabling a circular energy economy. Furthermore, as hydrogen can be stored and transported more easily than electricity, it presents an ultimate solution for balancing energy supply and demand across various sectors, making it a cornerstone of future sustainable energy strategies.

Electrochemical water splitting, which is composed of the cathodic hydrogen evolution reaction (HER) and the anodic oxygen evolution reaction (OER), is actually crucial for the efficient generation of green hydrogen, a clean and renewable fuel source [6]. While the HER is relatively straightforward, involving a 2e ^−^ transfer process, the OER is significantly more complex, requiring a 4e ^−^ transfer mechanism. This makes the OER the rate-limiting step in water oxidation, posing a major challenge to the overall efficiency of water-splitting systems [7,8]. The slow kinetics of the OER not only hinders the efficiency of hydrogen production but also leads to higher energy consumption, further complicating the commercial viability of large-scale water splitting. To increase the efficiency of the OER process, it is crucial to properly design electrocatalysts [9]. Noble metals such as Iridium (Ir) and Ruthenium (Ru) have long been recognized for their superior catalytic activity in OER, making them benchmarks for assessing the performance of new electrocatalysts [10]. However, their widespread commercial application is hampered by two major drawbacks: their high cost and their relatively limited long-term durability, which prevent them from being viable options for large-scale industrial use. Moreover, the scarcity of these noble metals further exacerbates their economic limitations, making large-scale deployment unsustainable [11,12]. As a result, developing OER electrocatalysts with high activity, strong durability, and low cost has attracted much attention from electrochemical researchers, with the goal of identifying alternative materials that offer similar performance at a fraction of the cost [13].

Extensive data confirms that transition metals, owing to their electronic structure, surface catalytic activity, stability, and ability to adopt multiple oxidation states, are highly suitable for electrochemical water oxidation, leading to the development of various transition metal-based electrocatalysts such as transition metal chalcogenides, nitrides, oxides, phosphides, hydroxides, and alloys [14,15,16,17,18]. These materials exhibit excellent catalytic properties that are crucial for promoting the oxygen evolution reaction (OER). However, unclear catalytic active sites and severe agglomeration issues hinder the practical application of transition metal-based electrocatalysts [19]. Selecting appropriate substrates can effectively provide clear catalytic active sites and prevent the aggregation of transition metals, which may be an effective solution [20]. Covalent organic frameworks (COFs) are functional crystalline porous materials constructed from pre-synthesized rigid organic molecules through appropriate condensation reactions, characterized by well-defined chemical structures, extremely high surface areas, strong stability, and designable pore size [21,22,23]. These unique structural properties allow for precise control over the incorporation of catalytic species, enhancing the overall efficiency of the electrocatalyst. In addition, COFs can be further functionalized to introduce heteroatoms as active components [24,25]. By the virtue of their unique advantages, COFs have the potential to serve as substrates for dispersing transition metals and providing well-defined catalytic active sites, applicable to electrocatalytic OER [26]. Banerjee and colleagues successfully designed a β-ketoenamine-linked COF, named TpBpy, which incorporates bipyridine units within its structure [27]. By further coordinating the framework with Co^2+^ ions, they developed a novel metal-coordinated COF, termed Co-TpBpy, for use as an electrocatalyst. The Co-TpBpy electrocatalyst exhibits an overpotential of 400 mV at a current density of 1 mA cm^−2^ when tested in a neutral phosphate buffer solution, demonstrating its promising catalytic activity in water oxidation reactions. This study highlights the potential of Co-TpBpy for efficient water oxidation in neutral conditions, making it a viable candidate for future applications in energy conversion and storage systems. 

In this study, a COF-IM with stable quinoid linkages and imidazole modifications was synthesized using TPB-DMTP-COF, which is connected via imine bonds, as the substrate [28], and pre-synthesized 2-ethynyl-1H-imidazole as the functional organic small molecule [29]. This innovative synthesis strategy takes full advantage of the flexible design possibilities inherent in covalent organic frameworks (COFs), while introducing imidazole groups that not only modify the COF structure but also impart catalytic functionalities. These imidazole modifications enhance interactions with transition metals, creating active coordination sites. The novel electrocatalysts (COF-IM@M) were prepared by coordinating the imidazole nitrogen atoms with transition metal ions, which further improves catalytic efficiency. Notably, COF-IM@Co, formed by coordinating with cobalt ions, demonstrated excellent electrocatalytic performance, exhibiting a low oxygen evolution reaction (OER) overpotential of 403.8 mV at a current density of 10 mA cm^−2^ in alkaline electrolytes. These findings highlight the potential of this method for designing efficient, metal-coordinated COF-based electrocatalysts, providing a promising avenue for future advancements in energy-conversion technologies.

## 2. Results

**Figure 1:**
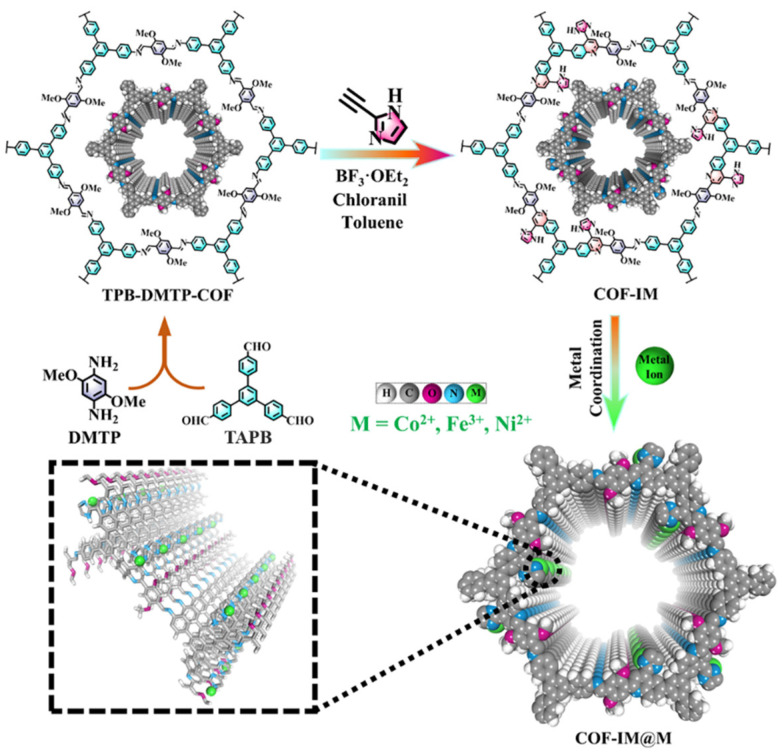
Schemes follow the same formatting. Schematic diagram of the synthesis process of TPB-DMTP-COF, COF-IM, and COF-IM@M.

The designed route for the fabrication of metal-coordinated COF-IM@M composites was shown in Figure 1. TPB-DMPT-COF was employed as a versatile substrate due to its facile preparation, stable structure, ease of post-synthetic modification, and outstanding specific surface area. These attributes make TPB-DMPT-COF an ideal candidate for hosting functional groups that can enhance catalytic activity. Subsequently, 2-ethynyl-1H-imidazole was immobilized on the channel surface of TPB-DMPT-COF through a Povarov cycloaddition reaction to prepare COF-IM. Given the strong chemical interactions between imidazole nitrogen and metal ions, metal ions (Fe^3+^, Co^2+^, and Ni^2+^) can be firmly anchored on the channels of COF-IM to synthesize metal COF-IM@M composites. This method not only increases the density of active sites but also ensures a more efficient electron transfer pathway, further enhancing the electrocatalytic performance of the resulting composites.

**Figure 2:**
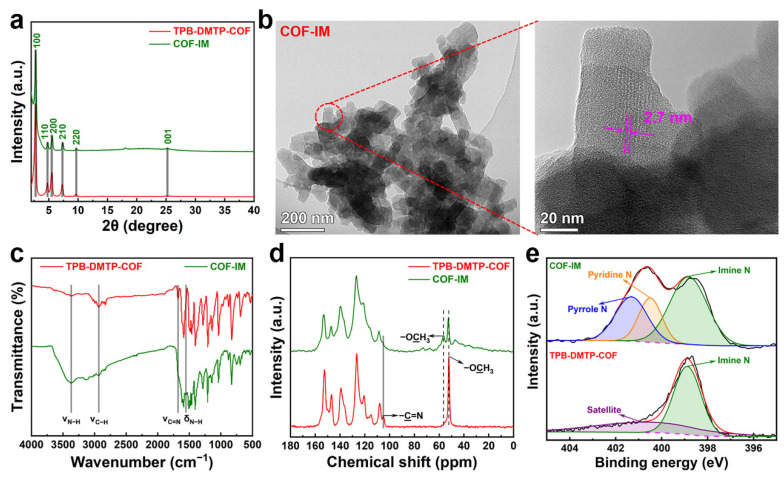
(**a**) PXRD patterns, (**b**) TEM and corresponding high-magnification TEM images of COF-IM, (**c)** FT-IR, (**d**) ^13^C CP/MAS NMR, and (**e**) N 1s XPS spectra for prepared TPB-DMTP-COF and COF-IM.

Powder X-ray diffraction (PXRD) analysis was conducted to determine the crystal structures of TPB-DMTP-COF, COF-IM, and COF-IM@Co, providing insight into the crystallinity and structural integrity of these materials. As depicted in Figure 2a, TPB-DMTP-COF displayed diffraction peaks at 2θ = 2.7°, 4.7°, 5.5°, 7.3°, 9.6°, and 25.6°, corresponding sequentially to the (100), (110), (200), (210), (220), and (001) crystal planes [30]. These observations illustrated that the TPB-DMTP-COF material possesses excellent crystallinity. The positions of the diffraction peaks for the imidazole-modified COF-IM and the further metal-coordinated COF-IM@Co remain nearly unchanged, indicating that the original crystal structure has not been significantly disrupted (Figure S1). Transmission electron microscopy (TEM) images of COF-IM reveal its clustered microstructure, with the high-magnification TEM image showing a distinct lattice spacing of 2.7 nm attributed to the (100) crystal plane (Figure 2b) [28]. Fourier transform infrared (FT-IR) spectroscopy was employed to reveal the chemical structures of TPB-DMTP-COF and COF-IM, with the corresponding spectra shown in Figure 2c. For both TPB-DMTP-COF and COF-IM, a prominent absorption band can be seen at 2940 cm^−1^, corresponding to the stretching vibration of saturated C-H bonds [31]. However, a key distinction between the two materials lies in the disappearance of the C=N stretching vibration at 1680 cm^−1^ in COF-IM, indicating the cleavage of imine bonds as a result of the post-synthetic modification [25]. This transformation is further confirmed by the appearance of a new absorption band at 3370 cm^−1^, corresponding to the N-H stretching vibrations, along with an additional peak at 1550 cm^−1^, associated with the N-H bending vibrations. These spectral changes confirm the successful modification of the COF structure with imidazole groups [31]. Furthermore, solid-state ^13^C cross-polarization/magic angle spinning (CP/MAS) nuclear magnetic resonance (NMR) spectroscopy provided additional insights into the structural differences between COF-IM and its precursor, TPB-DMTP-COF (Figure 2d). A notable observation is the disappearance of the characteristic signal at 104.9 ppm in the COF-IM spectrum, which is indicative of the degradation of imine carbons as a result of the cycloaddition reaction. In addition, the intensity of the methoxy carbon peak at 51.8 ppm decreases significantly following the imidazole modification. This is accompanied by the emergence of a new methoxy carbon peak at 56.4 ppm, suggesting a structural rearrangement during the functionalization process [28]. These results, taken together, offer strong evidence of the successful incorporation of imidazole groups into the COF framework, confirming the chemical transformation and modification of TPB-DMTP-COF into COF-IM. This comprehensive analysis demonstrates the structural integrity of the modified COF while highlighting the significant changes induced by the imidazole functionalization, which plays a crucial role in the material’s enhanced catalytic performance. The X-ray photoelectron spectra of TPB-DMTP-COF and COF-IM were obtained further to verify the successful post-synthesis modification (Figure 2e). The N 1s XPS spectrum of TPB-DMTP-COF only displays a signal peak for imine nitrogen at 398.9 eV [25]. In contrast, the N 1s XPS spectrum of COF-IM shows not only the imine nitrogen peak at 398.9 eV but also additional peaks attributed to pyridine nitrogen at 400.5 eV and pyrrole nitrogen at 401.3 eV. The results strongly support the transformation of imine groups, highlighting the successful incorporation of imidazole moieties into the COF structure. Based on this evidence, we can conclude that the conversion of imines to quinolines has been effectively achieved, along with the successful modification by imidazole units. These modifications provide a solid foundation for further structural and functional improvements of the COF materials.

**Figure 3:**
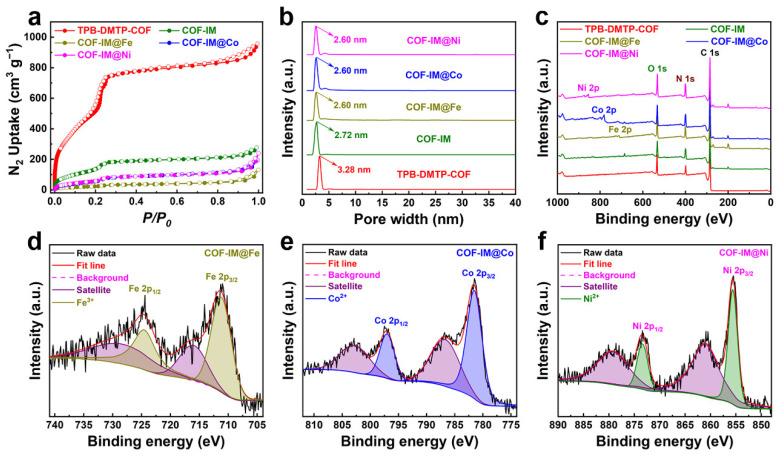
(**a**) Nitrogen sorption isotherm profiles, (**b**) pore size distribution curves, and (**c**) XPS survey scan of different COF-based materials; (**d**) Fe 2p XPS spectrum of COF-IM@Fe; (**e**) Co 2p XPS spectrum of COF-IM@Co; (**f**) Ni 2p XPS spectrum of COF-IM@Ni.

Meanwhile, we conducted nitrogen adsorption-desorption experiments at 77 K to investigate the specific surface area and pore size distribution of the samples before and after functionalization. Figure 3a shows that the original TPB-DMTP-COF, COF-IM, and COF-IM@Co exhibit Type IV nitrogen adsorption-desorption isotherms, indicating their mesoporous structural properties [32]. This mesoporosity is advantageous for enhancing the accessibility of active sites for catalytic reactions. Upon modification with the imidazole group, the BET surface area of TPB-DMTP-COF decreased significantly, dropping from 1990 m^2^ g^−1^ to 507 m^2^ g^−1^. Simultaneously, the uniform pore size was reduced from 3.28 nm to 2.72 nm. This trend continued upon further coordination with transition metals, where the BET surface areas of COF-IM@Fe, COF-IM@Co, and COF-IM@Ni decreased to 89, 222, and 226 m^2^ g^−1^, respectively, with corresponding uniform pore sizes decreasing to 2.60 nm (Figure 3b and Table 1). These changes in surface area and pore size are indicative of successful metal coordination and structural transformation. XPS survey scans provide further confirmation of the elemental composition of these materials. TPB-DMTP-COF and COF-IM contain C, N, and O, while the metal-coordinated materials—COF-IM@Fe, COF-IM@Co, and COF-IM@Ni—exhibit the presence of Fe, Co, and Ni, respectively, in addition to C, N, and O (Figure 3c). Detailed analysis of the XPS spectra reveals the oxidation states of the coordinated transition metals, with Fe in COF-IM@Fe existing as Fe^3+^, Co in COF-IM@Co as Co^2+^, and Ni in COF-IM@Ni as Ni^2+^ (Figure 3d–f). Energy dispersive spectroscopy (EDS) mapping further confirms the effective presence and uniform distribution of these transition metal elements within the COF structure, as shown in Figure S2. These findings highlight the successful incorporation and dispersion of metal ions, crucial for enhancing the material’s catalytic properties. This uniform distribution is critical for optimizing the electrocatalytic activity, as it ensures that the active sites are accessible for reaction with the substrate. Additionally, scanning electron microscopy (SEM) analysis reveals that COF-IM@Co exhibits a uniform bulk morphology (Figure S3). The above analysis shows that the nitrogen atom with higher electron density in the imidazole group can effectively interact with metal ions to form coordination bonds. The synthesized and modified quinoline ring exhibits strong coordination interactions with metal ions, enhancing the stability of the catalyst material. This improvement lays the foundation for COF-IM@Co to demonstrate excellent oxygen evolution reaction (OER) performance in alkaline electrolytes.

**Figure 4:**
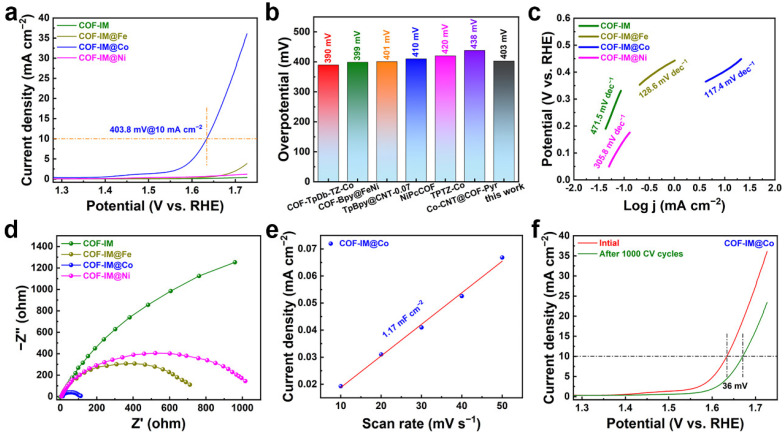
(**a**) LSV curves; (**b**) Comparison of overpotentials among different COF-based electrocatalysts in 1.0 M KOH electrolyte at a current density of 10 mA cm^−2^; (**c**) Tafel plots, and (**d**) Nyquist plots of COF-IM, COF-IM@Fe, COF-IM@Co, and COF-IM@Ni in 1.0 M KOH electrolyte; (**e**) C_dl_ value of COF-IM@Co; (**f**) LSV curves of the COF-IM@Co before and after 1000 CV cycles.

The controllable design of OER electrocatalysts is a current research hotspot, and we have also evaluated the OER activity of TPB-DMTP-COF, COF-IM, and COF-IM@M in a 1.0 M aqueous solution of KOH. For the linear sweep voltammetry (LSV), as depicted in Figure 4a and Figure S4, the COF-IM@Co with enhanced OER performance demonstrated an overpotential of 403.8 mV at 10 mA cm^−2^. This low overpotential indicates a significant improvement in catalytic efficiency, showcasing the effectiveness of the metal coordination strategy. Notably, the overpotential value for COF-IM@Co is lower than those of other reported COF-containing electrocatalysts (Figure 4b) [33,34,35,36,37,38]. For further perceptions of OER activity of the electrocatalysts, Tafel plots for COF-IM, COF-IM@Fe, COF-IM@Co, and COF-IM@Ni were collected from LSV curves. Tafel slopes were then obtained by linearly fitting these Tafel plots. The fitted Tafel slope for COF-IM@Co is 117.4 mV dec^−1^, which is significantly smaller than those for COF-IM (471.5 mV dec^−1^), COF-IM@Fe (128.6 mV dec^−1^), and COF-IM@Ni (305.8 mV dec^−1^), indicating that COF-IM@Co has faster reaction kinetics for OER (Figure 4c) [39]. This rapid kinetics suggests that COF-IM@Co not only enhances activity but may also improve the overall energy efficiency of the OER process. Nyquist plots of various electrocatalysts were acquired using electrochemical impedance spectroscopy (EIS) to investigate charge transport kinetics, and the corresponding results are shown in Figure 4d. Among the tested electrocatalysts, COF-IM@Co exhibited a significantly smaller curvature radius, indicating faster reaction kinetics and lower mass transfer resistance compared to its counterparts [40]. To further explore the source of its enhanced catalytic performance, the electrochemical surface area (ECSA) of COF-IM@Co was evaluated (Figure S5). Specifically, the double-layer capacitance (C_dl_) was measured at 1.17 mF cm^−2^, which suggests substantial intrinsic catalytic activity, as depicted in Figure 4e [41]. The enhanced ECSA observed in COF-IM@Co is a clear indication of an increased density of active sites, which significantly contributes to the improvement of its electrocatalytic efficiency. This increase in active sites facilitates more effective interaction with the electrolyte, promoting higher catalytic activity. The combined findings from EIS analysis and C_dl_ measurements underscore the superior charge transfer capabilities and robust catalytic performance of COF-IM@Co. These characteristics establish COF-IM@Co as a highly promising candidate for electrocatalytic applications in energy-conversion technologies. In addition to its remarkable catalytic activity, long-term cycling stability is a crucial factor for evaluating the practical viability of electrocatalysts. The durability of COF-IM@Co under extended operational conditions will be essential for its adoption in real-world applications. Thus, assessing its stability over prolonged cycles further strengthens its potential as an advanced electrocatalyst in sustainable energy systems. After 1000 CV cycles, the overpotential of COF-IM@Co increased slightly by 36 mV at a current density of 10 mA cm^−2^, demonstrating excellent retention of OER activity (Figure 4f). This minimal change in overpotential underscores the robustness of COF-IM@Co under prolonged operational conditions. SEM images of the catalyst after 1000 CV cycles revealed that the structure was maintained, further demonstrating the material’s excellent stability (Figure S6). In light of the results of the electrochemical tests, COF-IM@Co is an effective electrocatalyst for the OER electrocatalyst.

## 3. Experimental Section

### 3.1. Materials and Reagents

All materials and reagents involved in this work were sourced from reliable chemical companies and required no purification for direct use.1,3,5-tris(4-aminophenyl)benzene (TAPB) and 2,5-dimethoxyterephthalaldehyde (DMTA) were supplied from Jilin Chinese Academy of Sciences-Yanshen Technology Co., Ltd. Jilin, China. 2-imidazolecarbaldehyde, dimethyl (1-diazo-2-oxopropyl) phosphonate, and ferric chloride (FeCl_3_) were purchased from Shanghai McLean Biochemical Technology Co., Ltd. Shanghai, China. Cobalt chloride (CoCl_2_) and nickel chloride (NiCl_2_) were purchased from Shanghai Aladdin Biochemical Technology Co., Ltd. Shanghai, China. Other commercially available reagents were purchased in high purity and used without additional purification.

### 3.2. Characterization

PXRD patterns were collected using a Bruker (Billerica, MA, USA) AXS D8 Advance Labx diffractometer. FT-IR spectra were obtained using a PerkinElmer (Waltham, MA, USA) Spectrum 3 instrument. ^13^C CP/MAS NMR spectra were acquired on a Bruker (Rheinstetten, Germany) AVANCE III 500 MHz nuclear magnetic resonance spectrometer. XPS was performed using a Thermo Scientific (Waltham, MA, USA) ESCALAB 250Xi spectrometer. N_2_ adsorption-desorption measurements were conducted with a Micromeritics (Norcross, GA, USA) ASAP 2460 automated sorption analyzer. TEM images and energy-dispersive EDS mapping images were acquired using a Thermo Scientific (Waltham, MA, USA) Talos F200i S/TEM instrument.

### 3.3. Synthesis of TPB-DMTP-COF and 2-Ethynyl-1H-Imidazole

TAPB (56 mg, 0.16 mmol), DMTA (46 mg, 0.24 mmol), *o*-DCB (1 mL), *n*-butanol (1 mL), and HOAc (0.2 mL, 6 M) were sequentially added to a Pyrex tube. The mixture was dispersed using ultrasound for 10 min. The air in the Pyrex tube was removed by freeze-pump-thaw cycles, after which it was sealed by heating and maintained at 120 °C for 3 days. At last, the solid powder was washed with THF, purified using a THF-filled Soxhlet extractor, and dried under vacuum at 60 °C to acquire TPB-DMTP-COF [28].

2-Ethynyl-1H-imidazole was obtained based on the reported method, as demonstrated in Figure S7 [29]. To be more precise, 2-imidazolecarbaldehyde (0.668 g, 7 mmol), dimethyl (1-diazo-2-oxopropyl)phosphonate (1.996 g, 10 mmol), and K_2_CO_3_ (4.85 g, 35 mmol) were added to 40 mL of anhydrous methanol and stirred at room temperature for 72 h. After the reaction was complete, the mixture was concentrated under vacuum to half its volume and then extracted with 50 mL of ethyl acetate and 50 mL of saturated saline solution. The combined organic phase was subsequently dried over Na_2_SO_4_, and the solvent was removed under vacuum. The product was purified by column chromatography (silica gel; petroleum ether/ethyl acetate = 1:0 to 1:5), yielding a white powder of 2-ethynyl-1H-imidazole.

### 3.4. Preparation of COF-IM

In a Pyrex tube, TPB-DMTP-COF (0.2 mmol, 40 mg), 2-ethynyl-1H-imidazole (0.5 mol, 46 mg), chloranil (0.3 mmol, 80 mg), and toluene (4 mL) were successively added, followed by 10 min of ultrasound to achieve uniform dispersion. Subsequently, acid catalyst (20 μL of BF_3_∙Et_2_O) was introduced into the mixed system, and ultrasound was continued for another 10 min. The Pyrex tube underwent flame sealing and subsequent heating at a temperature of 110 °C for 3 consecutive days. Finally, the resultant precipitate was purified through suction filtration and subjected to multiple washes alternately with saturated NaHCO_3_, THF, and ultrapure water. After vacuum drying, COF-IM powder was acquired.

### 3.5. Fabrication of COF-IM@M

COF-IM@M composites were synthesized by dissolving COF-IM (50 mg) and a different metallic salt (50 mg) in 50 mL of dry methanol. Subsequently, the mixture was stirred under reflux at 60 °C for 10 h. The precipitate was purified by suction filtration and washing with dry methanol. Finally, the dark brown powder was dried at 60 °C for 12 h to obtain COF-IM@M composites.

### 3.6. Characterization Methods and Electrochemical Measurements

The OER properties of various electrocatalysts were investigated using a Gamry 1010E electrochemical workstation with a standard three-electrode system. The setup included the Hg/HgO electrode as the reference electrode, a graphite rod electrode as the counter electrode, a glassy carbon electrode as the working electrode, and a 1.0 M KOH aqueous solution as the electrolyte. The catalyst slurry was prepared by dissolving 5.0 mg of catalyst in 1 mL of a mixed solvent containing 95% ethanol and 5% Nafion, followed by ultrasonication for 30 min. The working electrode was prepared by adding 15 μL catalyst slurry to a glassy carbon electrode in three batches.

During OER testing, the electrocatalyst was first subjected to CV activation for 30 cycles with a scan rate of 100 mV s^−1^, and polarization data was collected using linear sweep voltammetry (LSV) measurements at a scan rate of 5 mV s^−1^. The electrochemical impedance spectroscopy (EIS) measurements were performed in frequency ranges from 100 kHz to 0.1 Hz at an overpotential of 403.8 mV. The double-layer capacitance (Cdl) was estimated by linearly fitting the current density plots at the same potential (1.23 V vs. RHE) with different scan rates from 10 to 50 mV s^−1^. Finally, the durability test of COF-IM@Co was carried out by the accelerated durability test (ADT), which cycled the potential from 1.28 to 1.73 V vs. RHE at 100 mV s^−1^ for 1000 cycles. 

## 4. Conclusions

In summary, based on the imine-linked TPB-DMTP-COF, we successfully synthesized the imidazole-modified COF-IM by introducing imidazole groups into the COF structure using a post-synthetic modification strategy and further coordinated it with transition metal ions (Fe^3+^, Co^2+^, and Ni^2+^) to obtain the novel COF-IM@M electrocatalysts. This innovative approach not only enhances the catalytic functionality but also leverages the unique properties of COFs to improve overall performance. Various analytical and testing methods thoroughly verified the successful synthesis of the new material and provided an in-depth investigation into its physicochemical properties. It is important to note that COF-IM@Co has been shown to be an effective OER electrocatalyst in alkaline electrolytes. The impressive performance of COF-IM@Co highlights the potential of using covalent organic frameworks as versatile platforms for the development of advanced electrocatalysts. This study provides a valuable strategy for the controlled design of high-performance electrocatalysts.

## Figures and Tables

**Table 1:** Porosity data of TPB-DMTP-COF, COF-IM, and COF-IM@M.

Samples	S_BET_^a^ [m^2^ g^−1^]	S_L_^b^ [m^2^ g^−1^]	V_total_ ^c^ [cm^3^ g^−1^]	D_pore_ ^d^ [nm]
TPB-DMTP-COF	1990	2148	1.48	3.28
COF-IM	507	480	0.43	2.72
COF-IM@Fe	89	83	0.20	2.60
COF-IM@Co	222	208	0.32	2.60
COF-IM@Ni	226	213	0.37	2.60

^a^ The specific surface areas were acquired via BET theory; ^b^ The specific surface areas were gained by Langmuir theoretical model; ^c^ Total pore volume at P/P_0_ = 0.99; ^d^ The pore sizes were obtained through the non-local density functional theory.

## Data Availability

Data will be made available on request.

## Supplementary Materials

The following supporting information can be downloaded at: https://www.mdpi.com/article/10.3390/molecules29215076/s1, Figure S1: PXRD curve of COF-IM@Co; Figure S2: HAADF-STEM image of COF-IM@Co and corresponding EDS mapping images of homogeneously distributed C, N, O, and Co (red bordered area); HAADF-STEM image of COF-IM@Fe and corresponding EDS mapping images of homogeneously distributed C, N, O, and Fe (green bordered area); HAADF-STEM image of COF-IM@Ni and corresponding EDS mapping images of homogeneously distributed C, N, O, and Ni (blue bordered area); Figure S3: SEM images of COF-IM@Co and corresponding EDS mapping images; Figure S4: LSV curve of TPB-DMTP-COF in 1.0 M KOH electrolyte; Figure S5: CV curves of COF-IM@Co at different scan rates from 10 to 50 mV s^−1^; Figure S6: SEM images of COF-IM@Co after 1000 CV cycles, along with the corresponding EDS mapping images; Figure S7: Synthesis route of 2-ethynyl-1H-imidazole; Figure S8: ^1^H NMR spectrum of 2-ethynyl-1H-imidazole; Figure S9: BET surface area plots of TPB-DMTP-COF; Figure S10: BET surface area plots of COF-IM; Figure S11: BET surface area plots of COF-IM@Co; Figure S12: BET surface area plots of COF-IM@Fe; Figure S13: BET surface area plots of COF-IM@Ni. Reference [42] is cited in the supplementary materials.
