# Supplementary material for: Metal Imidazole-Modified Covalent Organic Frameworks as Electrocatalysts for Alkaline Oxygen Evolution Reaction
Source: Molecules. 2024 Oct 27;29(21):5076. doi: 10.3390/molecules29215076 (PMC11547209; doi:10.3390/molecules29215076)
Supplement: Supplementary file 1 [file molecules-29-05076-s001.zip › molecules-3247725-supplementary.pdf]

Supporting Information

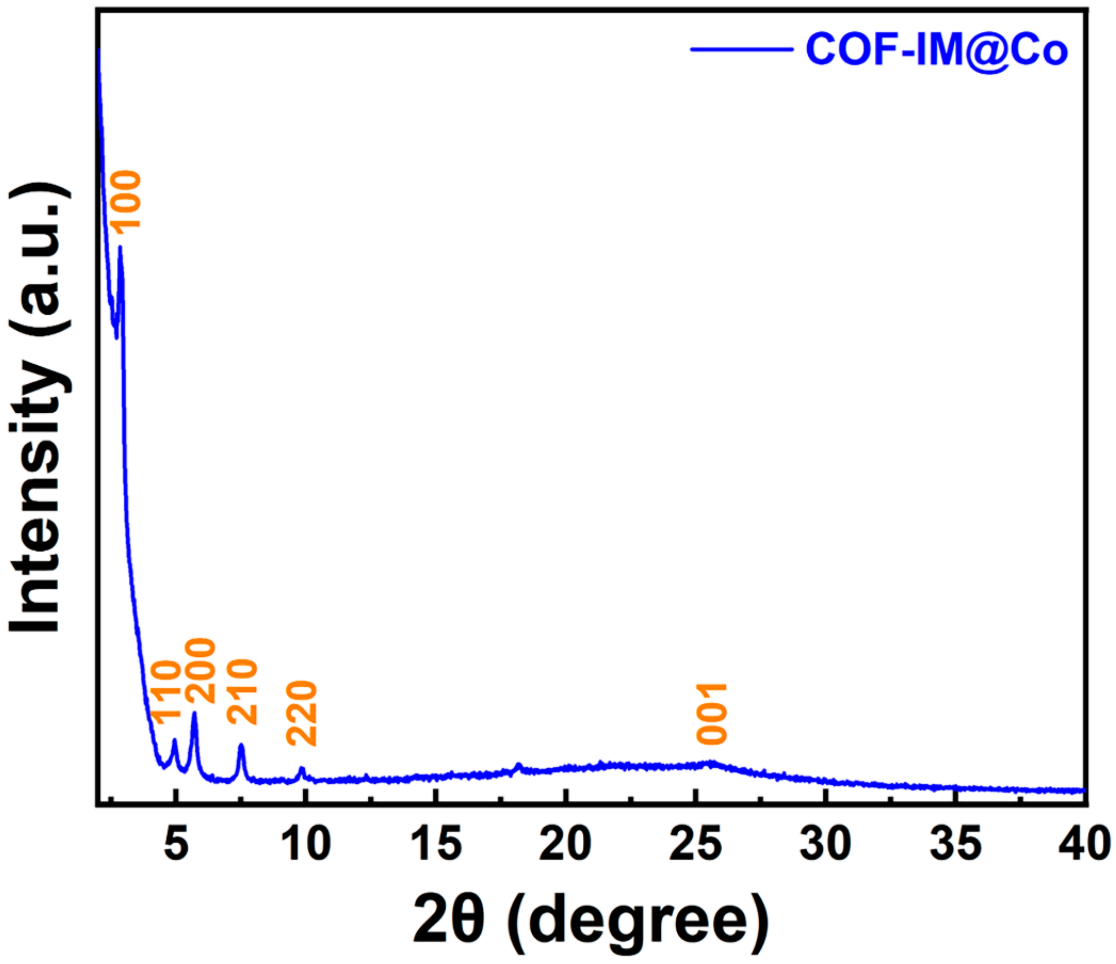

Figure S1. PXRD curve of COF-IM@Co.

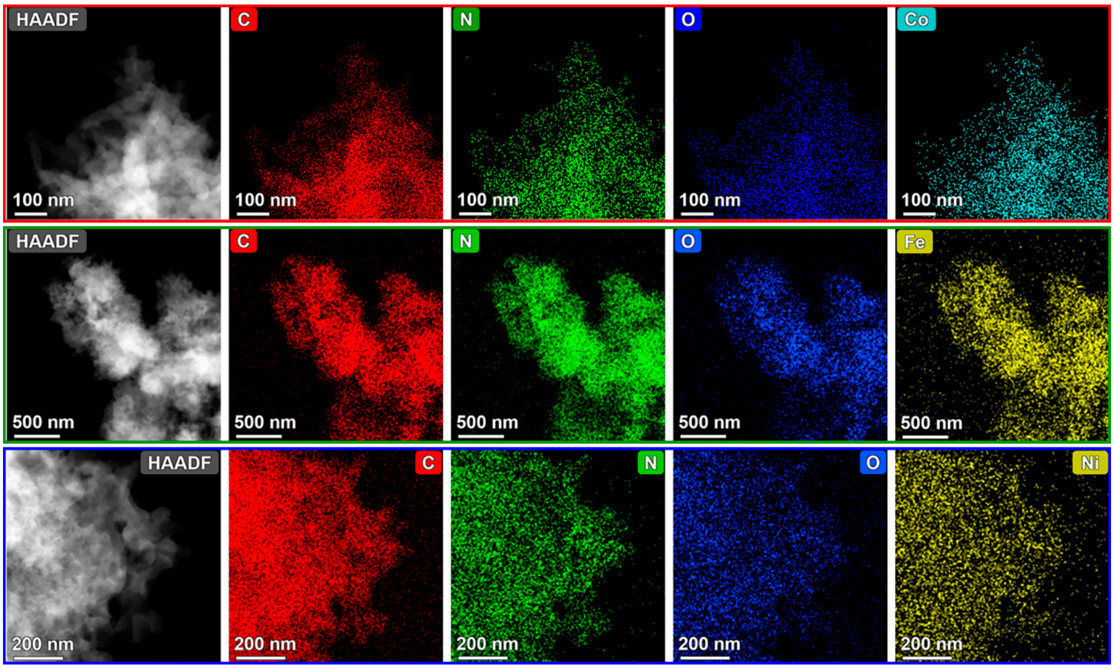

Figure S2. HAADF-STEM image of COF-IM@Co and corresponding EDS mapping images of homogeneously distributed C, N, O, and Co (red bordered area); HAADF-STEM image of COF-IM@Fe and corresponding EDS mapping images of homogeneously distributed C, N, O, and Fe (green bordered area); HAADF-STEM image of COF-IM@Ni and corresponding EDS mapping images of homogeneously distributed C, N, O, and Ni (blue bordered area).

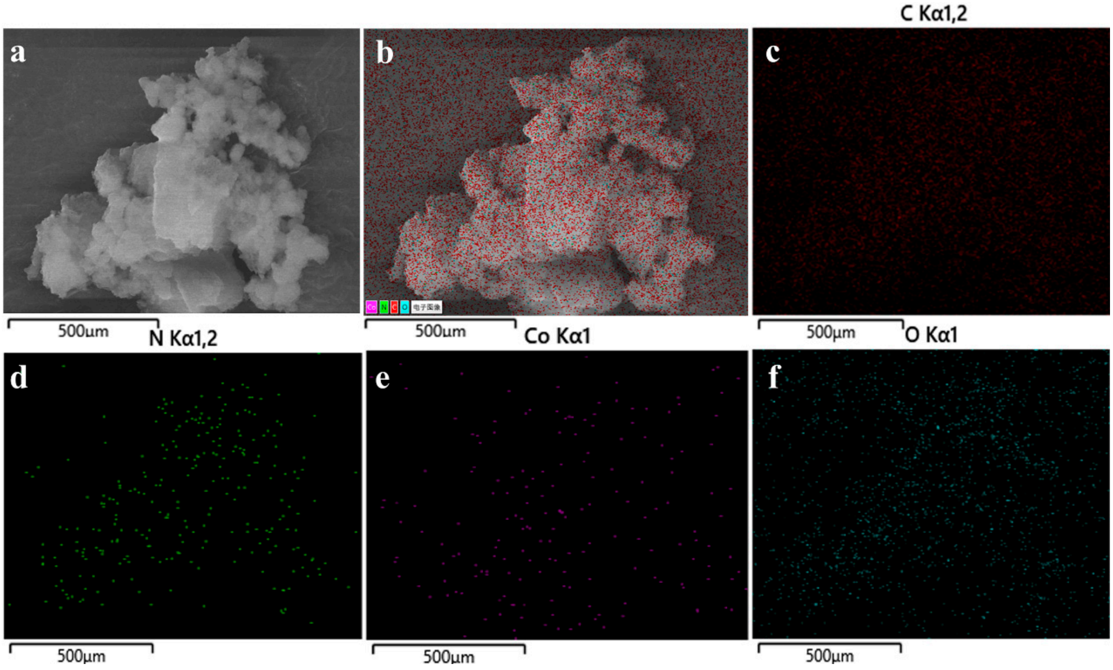

Figure S3. SEM images of COF-IM@Co and corresponding EDS mapping images.

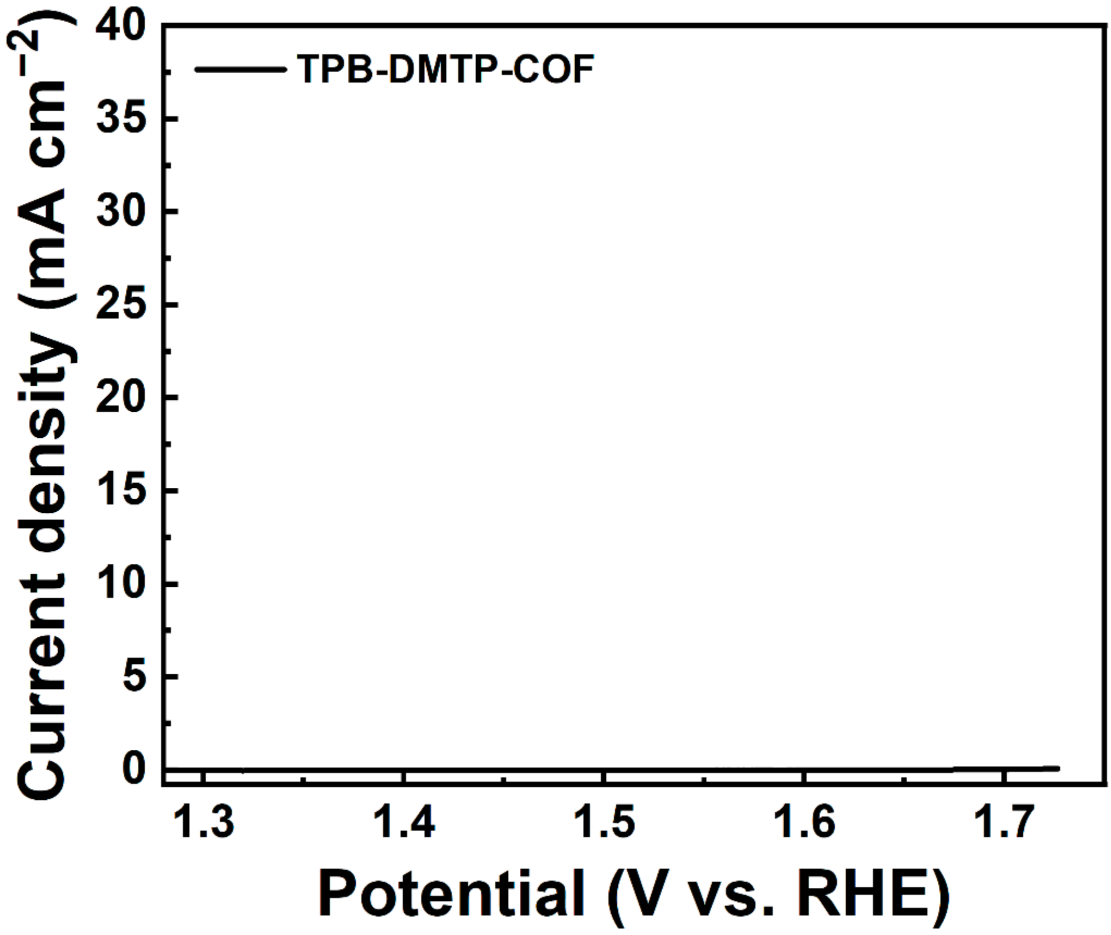

Figure. S4. LSV curve of TPB-DMTP-COF in 1.0 M KOH electrolyte.

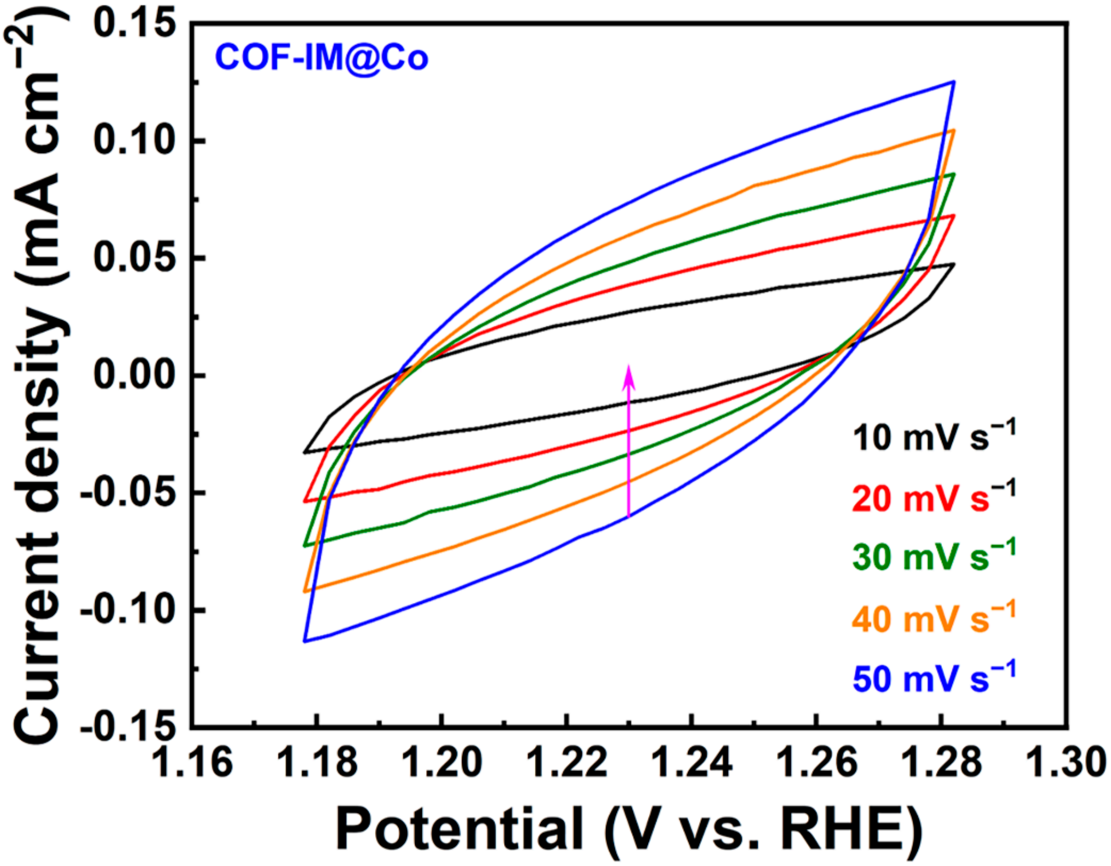

Figure S5. CV curves of COF-IM@Co at different scan rates from 10 to 50 mV s^{-1}.

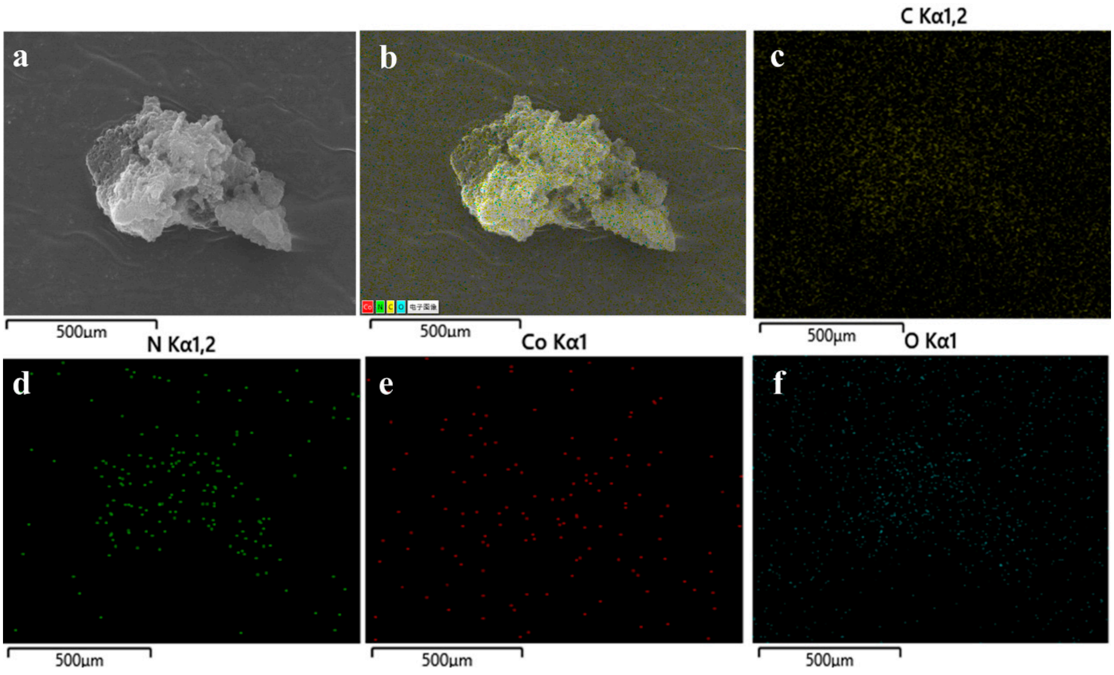

Figure S6. SEM images of COF-IM@Co after 1000 CV cycles, along with the corresponding EDS mapping images.

1. Synthesis of 2-ethynyl-1H-imidazole

The synthesis of 2-ethynyl-1H-imidazole was conducted systematically, incorporating minor modifications based on method reported in the literature (Figure S1) [S1, S2].

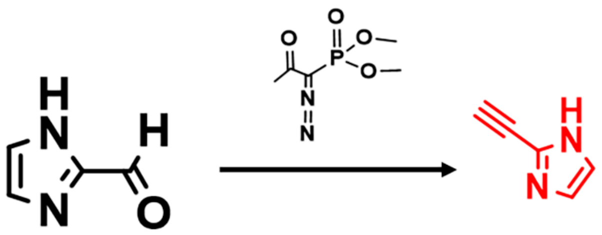

Figure S7. Synthesis route of 2-ethynyl-1H-imidazole.

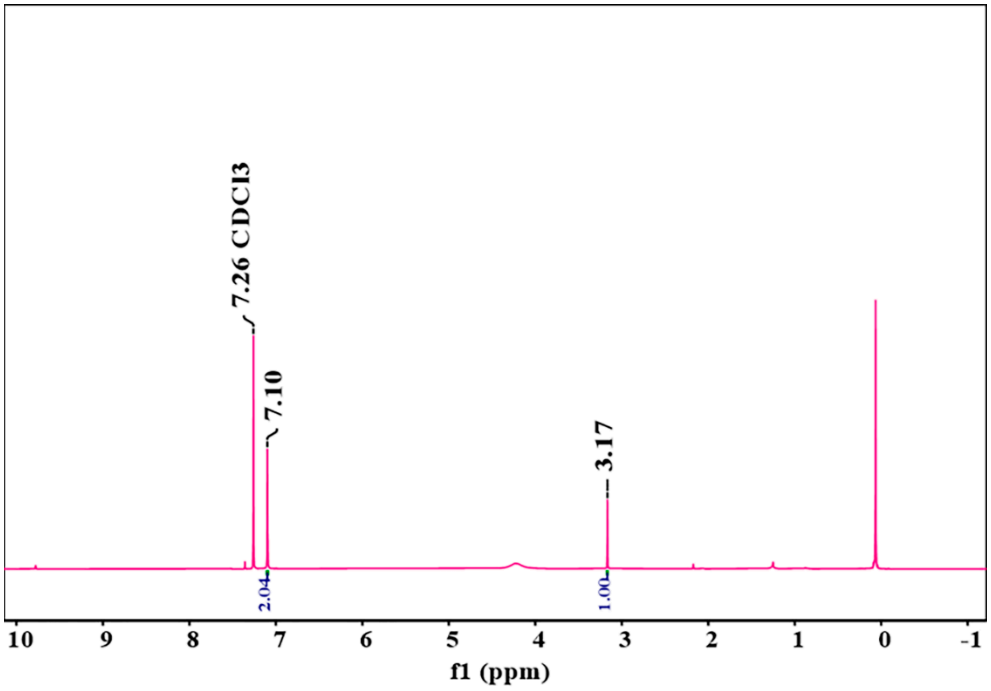

Figure S8. ^{1}H NMR spectrum of 2-ethynyl-1H-imidazole.

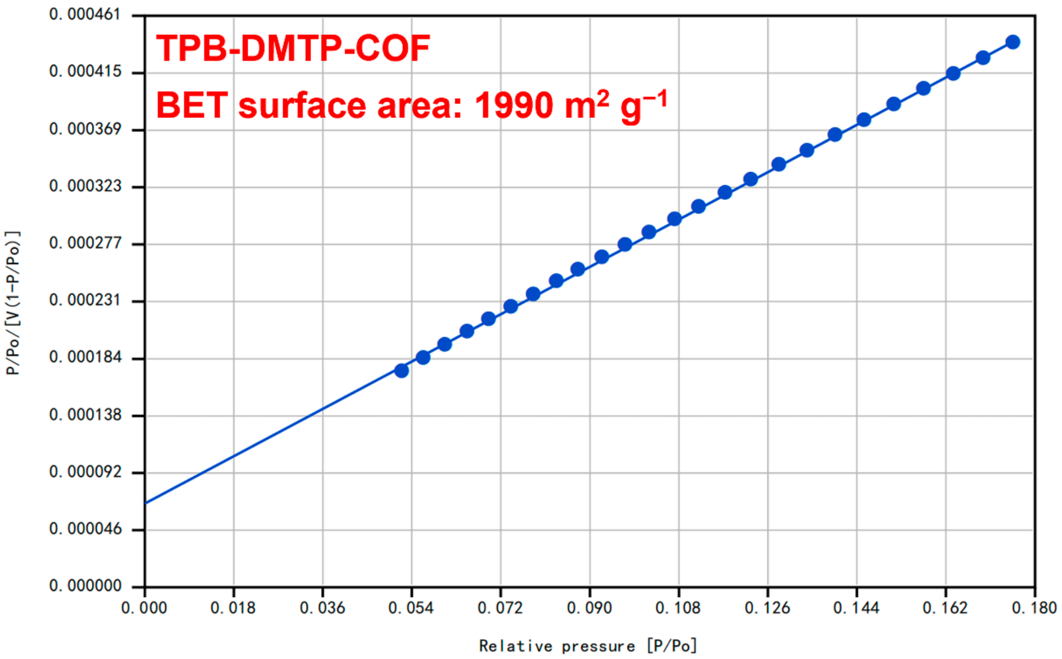

Figure S9. BET surface area plots of TPB-DMTP-COF.

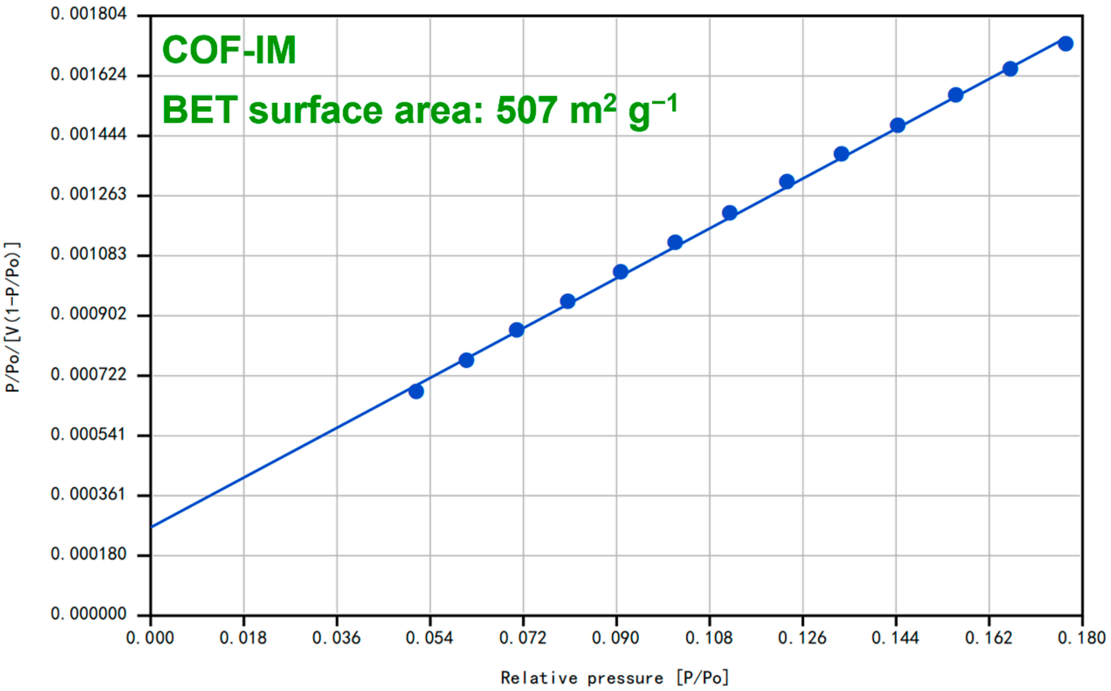

Figure S10. BET surface area plots of COF-IM.

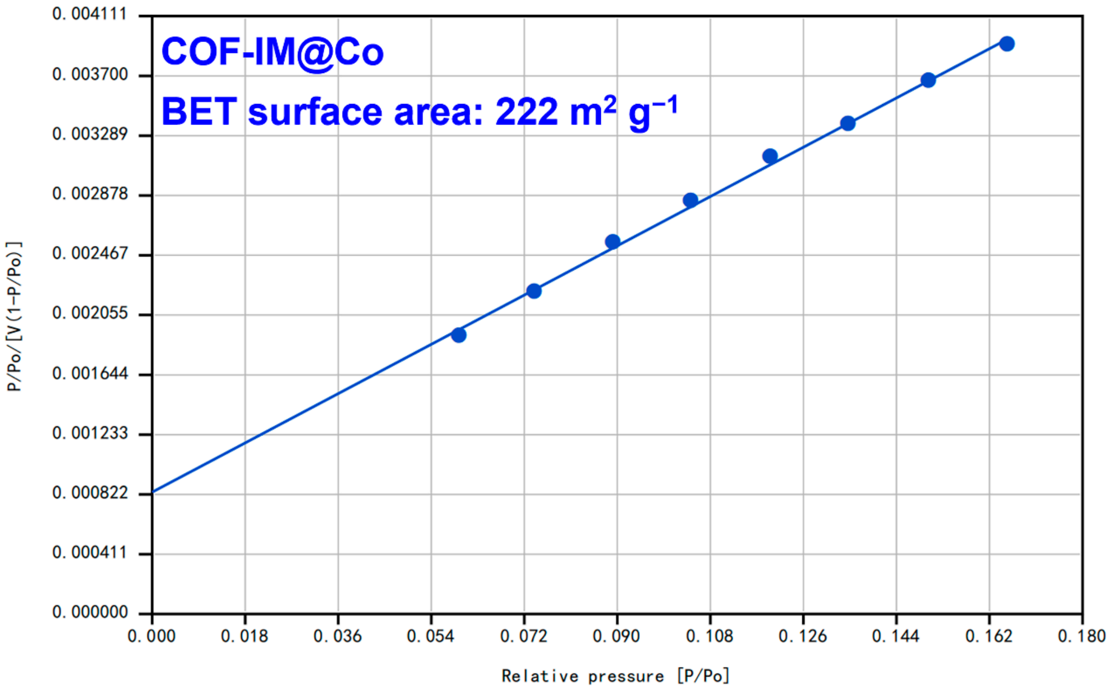

Figure S11. BET surface area plots of COF-IM@Co.

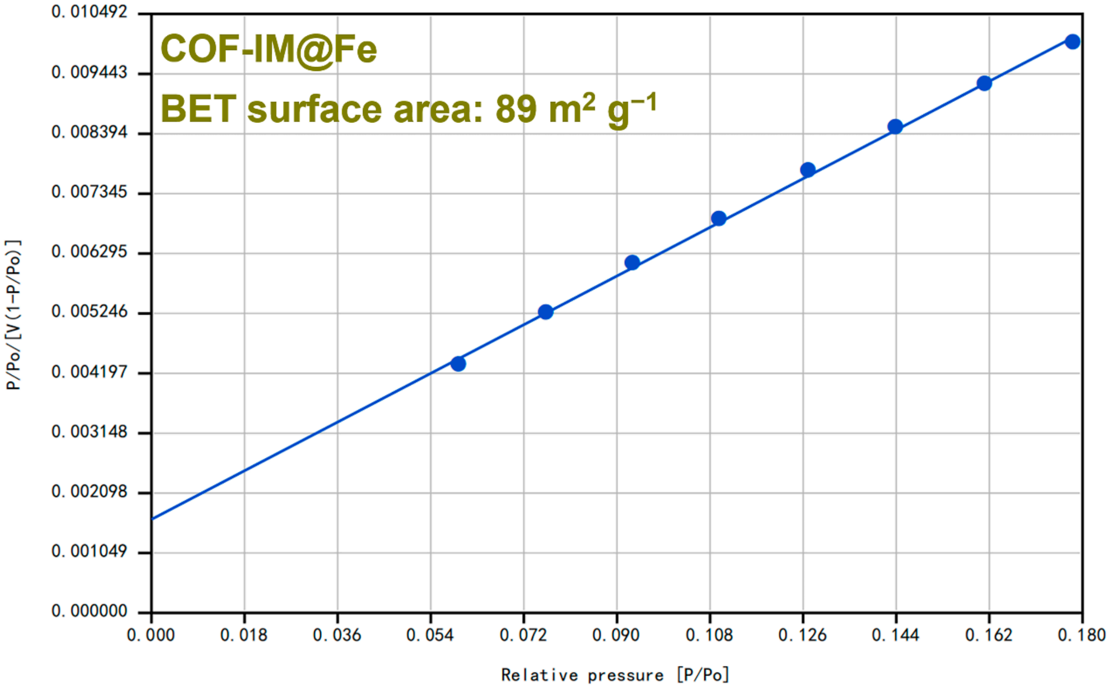

Figure S12. BET surface area plots of COF-IM@Fe.

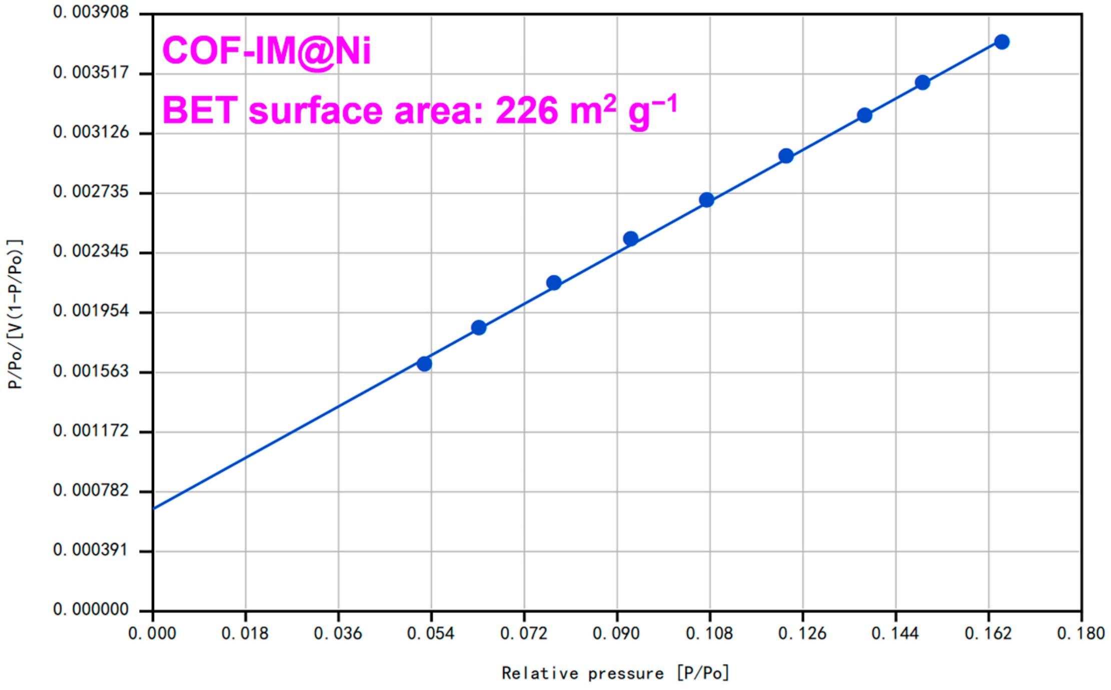

Figure S13. BET surface area plots of COF-IM@Ni.

References

[S1] Rauch V., Wytko J. A., Takahashi M., Kikkawa Y., Kanesato M., Weiss J., Dynamic assembly of porphyrin wires trapped on a highly oriented pyrolitic graphite surface. *Org. Lett*., **2012**, 14:1998−2001.

[S2] Roth G. J., Liepold B., Müller S. G., Bestmann H. J., Further improvements of the synthesis of alkynes from aldehydes. *Synthesis,* **2004**, 1:59−62.
